# Cases of Injuries of the Head

**Published:** 1827-04

**Authors:** C. Bell

**Affiliations:** *the* Middlesex Hospital.; *the* Middlesex Hospital.; *the* Middlesex Hospital.


### INJURIES OF THE HEAD.

*Cases of Injuries of the Head, treated by* Mr. Joberns, Mr. C. Bell, *and* Mr. Shaw, *at the* Middlesex Hospital.

(Continued from page 246.)

### *Contusions of the Skull.*

Contusion of the skull, the effects of which have been so admirably described by Pott, is generally the consequence of a smart blow received on the head from some blunt instrument,—as the flat end of a hammer or a cudgel. Such a blow is very apt to separate the dura mater from the internal surface of the bone; to destroy the connexion of the pericranium with the skull; or so to injure the vessels of the bone as to render them unfit for its further support and nourishment. The bone dies, becomes a source of irritation to the brain, and inflammation with its consequences supervene.

Case X. *Contusion of the Skull, followed by Inflammation and Abscess in the Brain.* Treated by Mr. Shaw.

Patrick Doyle, æt. twenty-seven, a strong Irish labourer, was brought to the hospital, about half-past six o'clock, on the evening of the 25th of October, 1825. He was perfectly insensible; his breathing was stertorous, and the pupils of the eyes were dilated; the pulse was rather full;—in short, his symptoms were exactly those of a man in a deep apoplexy.

It appeared that, about fifteen or sixteen days before, this man had been drinking with some women of the town, in a public-house in St. Giles's. His wife, having received intelligence of this, entered the room unobserved, and suddenly assailed him with a half-gallon tin can, with which she struck him several times on the head. A blow on the forehead felled him to the ground, where he lay weltering in blood. After this affray he was conveyed home, and in the space of four days he found himself so far recovered as to be able to resume his usual occupations. In a few days, however, he was obliged to betake himself again to his bed, so much did he suffer from violent pain in the head. He lay in this condition for more than a week, and his constant request was "that some one would bind up his head tight." At the end of this period, we are told that he "became heavy for sleep;" that he gradually lost all consciousness, and at length fell into a complete state of insensibility. In this condition he was taken to St. Giles's workhouse, where he remained for two days; and from thence he was conveyed to the hospital. It was afterwards elicited at the inquest that this man had also been struck several blows on the head with a hammer.

The above history was extracted from the friends with the utmost difficulty: they appeared unwilling to give any information; so that the accounts which were at first received were so discordant and unsatisfactory, that the surgeon was obliged to depend entirely upon the symptoms in forming his diagnosis.

The head was examined, and numerous cicatrices were found on the scalp. There was a wound on the forehead, about two inches above the right orbit: here a probe was introduced, and the bone was found to be bare, its surface rough and irregular, insomuch that the sensation communicated to the hand gave rise to the idea that the bone was fractured. Mr. Shaw enlarged the wound of the scalp, and thus disclosed a circular portion of bone, a quarter of an inch in diameter; it was denuded of its periosteum, of a greyish-white colour, and apparently dead. This small piece of bone appeared to be indented; but this deceptive appearance was caused by a thickening of the pericranium around the dead bone. The process of exfoliation had begun.

This was certainly a hopeless case. The symptoms were evidently those which accompany an oppressed state of brain; and their progress and duration clearly pointed out that such oppression must arise from a collection of pus or serum within the cranial cavity, interrupting the due circulation of blood through the brain. The only chance, therefore, of affording relief was in the removal of the dead portion of bone, in the hope that the cause of oppression might be found between the skull and dura mater.

The wound of the scalp was enlarged, and the trephine was set on in such a manner as to include the dead bone. The patient, during the operation, gave no signs of sensibility. The dura mater was observed to be a little discoloured; it was thinly covered with a sero-purulent fluid. The portion of bone removed was dead throughout; its internal surface was rough, and of a darker colour than the surrounding bone. The dura mater was covered, as usual, with circular pieces of oiled lint; the integuments were laid down, and gentle support was afforded by the application of a roller. The operation was, as had been feared, quite ineffectual, and the man died in about three hours.

*Dissection.*—The skull was cut in the usual manner for examining the head; but the surgeon, expecting to find an abscess in the cerebrum, carried his knife horizontally through the dura mater and hemispheres of the brain, on the same level with the division of the skull. Thus the hemispheres of the cerebrum were removed with the skullcap. A large quantity of serous fluid was found in the ventricles. There was an abscess in the right hemisphere of the cerebrum, at the part situated immediately beneath the dead portion of bone. Here also the surface of the brain was discoloured, and adherent to the internal surface of the dura mater. The parts of the brain surrounding the abscess were much softened, and in this softened texture spots of extravasated blood were very numerous. No other morbid appearances presented themselves.

Case XI. *Contusion of the Cranium, in which the Bone exfoliated.* Treated by Mr. Joberns.

Mary Braggen, æt. seven, admitted September 18th, 1826. This child had fallen from a window on the first floor: when picked up, she was perfectly sensible, and was brought immediately to the hospital. There was no aberration of intellect or want of consciousness, to mark the presence of concussion; but the influence of its more partial effects might be observed in the pallid countenance, the small and weak pulse, and the disturbed condition of the stomach, indicated by frequent vomiting. There was a wound of the scalp, three inches in length: it commenced near the root of the nose, took a direction upwards, traversed the upper part of the forehead, and then descended towards the left temple; so that a semicircular flap of the integument, covering the left side of the forehead, was detached from the bone. The wound was of a contused character, and the cellular membrane was filled with mud. The skull was to some extent deprived of its periosteum, and a portion of the cranium, nearly two inches in circumference, was beaten in from its centre. There was no abrupt or broken edge of bone; it formed a regularly concave depression.

When the bleeding had ceased, the edges of the wound were brought together by adhesive straps, and the part was kept cool by an evaporating lotion. She was well purged, and an antimonial mixture was afterwards prescribed. In the evening, the pulse was full and quick, and the skin was hot. Leeches were applied to the head.

For the first three days, symptoms of inordinate vascular action of the brain were present, which were met by appropriate antiphlogistic measures. The contused nature of the wound prevented union by the first intention; the wound became sloughy, and discharged a dark coloured and thin sanies. Long adhesive straps were applied, in order to prevent that retraction of the integuments which is so apt to occur in wounds of the scalp; and the wound was covered with a poultice of bread and water.

October 1st.—The wound has healed, except at the part situated over the contused bone: this also contracts, so that we have reason to hope that the bone may not be very extensively destroyed.

Portions of bone afterwards came away by a tedious process of exfoliation.

### *Fractures of the Skull.*

Before relating any cases of fracture of the skull, we cannot do better than repeat the sentiments contained in the first sentence of this series of cases. Thus, in the language of Mr. John Bell, we would say, "that, to think that a fractured skull is a chief cause, or even an absolute sign of danger, is a very poor and vulgar notion: it is not the damage done to the skull, but the injury to the brain, that is the cause of danger, and the fracture of the skull is but a faint and uncertain mark of the harm done to the brain."

Case XII. *Compound Fracture of the Skull, with Rupture of the Dura Mater, followed by Fungus Cerebri.* Treated by Mr. Joberns.

Charles Shuggate, æt. thirteen, was brought to the hospital June 26th, 1826, at nine P.M. He had received a kick on the temple from a horse; he remained insensible for a few minutes. There was a wound about three inches in length, which commenced a little anterior to the right emminentia frontalis, and, proceeding backwards, terminated at the superior margin of the temporal muscle. The finger was introduced, and the skull was found to be fractured. The fracture ran in the same direction as the wound, crossed the coronal suture, and thus included both the frontal and right parietal bones. The portion of bone above the line of the fracture was depressed about a quarter of an inch below the level of the surrounding bone. The depressed piece of bone was nearly three inches in length. The dura mater was ruptured, as shown by the discharge of brain and blood by the wound. Some attempts were made to elevate the depressed bone; but, as this was found to be impracticable without removing a part of the skull, and as the boy was quite sensible, roaring loudly, with a pulse at ninety, and beating quickly, the opening was not enlarged; the bone, although detached from the skull, was left lying on the dura mater; it being considered less likely to cause a dangerous inflammation than the high excitement which would necessarily be the consequence of an operation under the existing circumstances.

The wound was left open, to allow of a free escape of blood, &c. The head was shaved, and covered with an evaporating lotion; and some aperient medicine was administered.

27th.—He was extremely quiet this morning. He did not complain of any pain in the head, except in the situation of the wound. Pulse ninety-five; tongue clean and moist; skin cool.

The bowels not having been acted upon, he was ordered a purgative enema; and the following draught was directed to be taken every six hours—Magnesiæ Sulph. ʒj.; Vin. Antim. Tart. m. xx.; Aquæ distillat. ℥j.

28th.—He complained of slight headache. The countenance was dull, and there was a dropping of the upper eyelids; the tongue was rather dry, and was traversed by a brownish central line; skin hot; pulse eighty-eight.

Nine ounces of blood were taken from the arm, when the pulse began to faulter.—To continue taking the Antimonial Mixture, with a smaller quantity of Sulphate of Magnesia.

29th.—The abstraction of blood appears to have been attended with the happiest effects. He is free from pain in the head; but he complains of a throbbing sensation in the situation of the wound. The skin is cool and perspiring; tongue moist and less furred; pulse sixty-eight, and soft. The wound has been poulticed for the last two days; it still continues to discharge brain and matter.

July 2d.—No change till to-day. Again he began to complain of pain in his head. Pulse seventy-six, strong, and cord-like.

He was bled to ten ounces.—Calomel gr. iv.; Opii gr. ss. to be taken at bedtime.

3d.—He expressed himself much relieved by the bleeding. Pulse seventy-two, much softer; tongue moist, and covered with a thin and yellow fur. A small, pulsating, and brain-like protuberance was observed in the wound: this substance was similar to an incipient fungus cerebri.

6th.—There had been a diminution in the severity of all the symptoms since the last report; but, on visiting him this morning, we found his skin hot, his tongue dry, and the pulse quick and corded. The pupils were dilated, and he slumbers much. The protrusion from the brain has now assumed all the characters of Fungus Cerebri. It was a pulsating tumor, as large as a pigeon's egg, of a greyish colour; its surface granulated, and here and there covered with small spots of coagulated blood.

A piece of simple dressing was placed on the tumor, and slight pressure maintained by adhesive straps.

8th.—He is inattentive to questions, and his answers are incoherent. His eyelids are constantly closed; the pupils are dilated; and the pulse is slow and intermitting. A probe was introduced by the side of the tumor, but there was no confined matter.

10th.—Restless and noisy, and tore the dressings from the wounds. Ten leeches were applied to the temples.

12th.—During the whole of yesterday he was much quieter, and appeared to be relieved; but to-day the report is not so favourable. He had passed his feces and urine in bed; pulse fifty-five, and intermittent.

Hydr. Submur. gr. ss.; Pulv. Antim. gr. j. to be taken three times a-day.

There has been no increase of the fungus since the application of the adhesive straps.

14th.—The fungus has began to slough, and there is no disposition to a fresh protrusion. He appears to be less comatose. Pulse still slow, and somewhat hard.

17th.—He no longer passes his feces and urine in bed. He says that the throbbing sensation which he has felt from the first in the situation of the fungus is much less. He raises the eyelids with more facility. The wound continues to discharge a large quantity of pus.

20th.—His improvement is now rapid: he is much more sensible; there is no appearance of fungus remaining; the wound is granulating kindly, but the discharge is still profuse. Pulse sixty; tongue clean and moist.

He had a slight attack of fever on the 23d, after which period the progress of amendment was uninterrupted. He was detained in the hospital for several months, on account of an exfoliation of the bone. He left the hospital with the bone as much depressed as at first.

This wound of the brain was in the situation of the organ of *gaiety*, but, from what we can learn from the friends, he exhibits no deficiency in this respect, being as merry as before.

Case XIII. *Extensive Compound Fracture of the Skull.* Treated by Mr. Bell.

James Barnes, æt. five, admitted into the hospital September 20th, 1826. He had fallen head-foremost into a cellar: he was senseless for about a quarter of an hour. There was a small wound of the scalp covering the anterior and lower parts of the right parietal bone, so small as not to admit of the introduction of the little finger. With the assistance of the probe, the skull was discovered to be fractured. The fracture extended backwards in a semicircular form, taking much the same course as the squamous suture; and, inasmuch as it was an inch above this suture, so was it of much greater extent. The upper portion of the bone was depressed about a quarter of an inch below the lower or temporal part of the skull. Here, then, was a great part of one side of the skull beaten in and depressed. The child was, at the time it was brought to the hospital, suffering from most of the important effects of concussion. The skin was cold, the pulse small, and he vomited frequently when the bleeding had ceased. The usual means were adopted of uniting the wound, and cold was applied to the head.—In the evening, reaction had taken place; the pulse was full and strong, the skin hot and dry. Ten leeches were applied to the head, and he was ordered to take a brisk purgative.

21st.—There was great tenderness and tumefaction of the scalp on the injured side. The heat of skin, and other febrile symptoms, were much diminished; but the marks of a violent commotion of the brain were still present,—the pale and anxious countenance remained unchanged, and the vomiting continued.

23d.—To-day the scalp was hot; the tongue slightly furred, and the pulse hard and strong. He appeared to be averse to light. In consequence of the vomiting continuing unchecked, twelve leeches were applied to the head; and, to allay this irritability of the stomach, which was originally only symptomatic, the following draught was ordered to be taken every six hours—

H. Salin. ℥j.; Vin. Antim. Tart. m. xv.; Træ. Opii m. iij.

26th.—The vomiting did not recur after the second dose of the medicine had been given; so that, on the following day, the tincture of opium was omitted, and the antimonial mixture only was continued.

No unfavourable symptoms occurred after this. The wound suppurated in the whole length of the fracture, and it was found necessary to make a counter opening behind the ear. The matter, when collected under the scalp, receives a distinct impulse as often as the child cries or coughs: on the other hand, when the child sobs, or otherwise draws the air into the lungs, the abscess is, as it were, emptied of its fluid contents, and the tumor of the scalp subsides. This pulsatory motion, which is communicated to the matter of the abscess, must be evidently produced by the matter lodging upon the dura mater.

October 14th.—The cavity of the abscess has diminished in size, much of the surrounding scalp having adhered and become attached to the bone. The health is good: in short, things wear a most favourable aspect. He was discharged.

A small scale of bone was afterwards thrown off.

Case XIV. *Compound Fracture of the Skull.* Treated by Mr. Shaw.

Caroline Free, æt. four, was admitted on the 12th June, 1826. She had fallen on her head from a height of thirteen feet. When picked up, she was senseless, and remained so for some minutes. There was a wound of the scalp, of an inch and a half in length, over the coronal suture on the left side, and just above the temporal muscle. The finger was introduced, the skull was felt to be fractured and depressed. The portion depressed might be somewhat more than an inch in length, and was about a quarter of an inch below the level of the surrounding bone. There was not one unfavourable symptom present, further than that the pulse was rather weak, and the child's countenance somewhat paler than usual. Having ascertained that the depressed piece of bone was not sharp or angular, the wound was united by adhesive straps.

The favourable progress of this case was uninterrupted. No symptoms of oppression occurred; and the slight disposition to inflammation which followed was easily controlled by the exhibition of calomel, antimony, and purgatives. She was discharged before the end of June.

The preceding cases may be regarded as good examples of the little interference required on the part of the surgeon in elevating depressions of the skull in children. "Perhaps there is no rule in surgery more correct in theory, nor better supported by authority, than that which warns the surgeon to beware of being too busy in raising the depressions of the skulls of boys." "Where depressed bone has in general no sharp edges,—where the skull rather bends than breaks,—where the bone is vascular and growing, and the circulation in it and in the integuments sound and vigorous, the chance of fracture healing is so great that I should not presume to touch it, unless in most particular circumstances." Yet has it been laid down by some, that every compound fracture of the skull with depression should be trephined, whether symptoms of compression be present or not. But, when no symptoms are present, we would rather say, that, "whenever a piece of bone is so isolated or separated from its connexions that it must die, it should be taken away; for it produces exactly the same effect on the dura mater, and ultimately upon the pia mater, as if a foreign body lay there. When, from the form of the fractured bone, (judging from what is external,) you conclude that a sharp angle or edge presses in upon the membranes, the depressed portion should be raised, if not entirely removed."

